# Pediatric exogenous lipoid pneumonia caused by paraffin oil aspiration: a case report

**DOI:** 10.3389/fped.2026.1929480

**Published:** 2026-09-23

**Authors:** Dingbo Tian, Bayu Nie, Junhong Duan

**Affiliations:** 1 Department of Pediatric Critical Care Medicine, Children’s Medical Center, People's Hospital of Xiangxi Autonomous Prefecture, The First Affiliated Hospital of Jishou University, Clinical College of Jishou University, Xiangxi, Hunan, China; 2 Pediatric Emergency, Children’s Medical Center, People's Hospital of Xiangxi Autonomous Prefecture, The First Affiliated Hospital of Jishou University, Clinical College of Jishou University, Xiangxi, Hunan, China; 3 Department of Radiology, The Third Xiangya Hospital of Central South University, Changsha, Hunan, China

**Keywords:** aspiration, exogenous lipid, lipoid pneumonia, paraffin oil, pediatric

## Abstract

**Objective:**

This case report describes a rare pediatric case of exogenous lipoid pneumonia caused by repeated paraffin oil aspiration and highlights the importance of detailed exposure history, early recognition, and timely intervention.

**Case presentation:**

We report the clinical course, diagnostic assessment, treatment, and follow-up of a 4-month-old former preterm infant admitted to the pediatric intensive care unit with severe pneumonia.

**Results:**

Repeated targeted history-taking revealed multiple oral administrations of liquid paraffin with choking episodes. Chest CT showed bilateral consolidation, ground-glass opacities, interlobular septal thickening, a crazy-paving pattern, and a focal area of fat attenuation. BALF cytology showed vacuolated histiocytes, although lipid-specific staining was unavailable. *Klebsiella aerogenes* was detected by throat-swab and BALF targeted next-generation sequencing and was isolated from sputum culture, supporting concomitant lower respiratory tract infection. Following discontinuation of paraffin exposure and multimodal management, the patient showed clinical and radiological improvement.

**Conclusion:**

Exogenous lipoid pneumonia should be considered in infants with treatment-refractory pneumonia and intralesional fat attenuation on CT. Diagnosis requires integration of exposure history, imaging, BALF findings, microbiological evidence, and clinical course. Because several interventions were administered concurrently and long-term follow-up was incomplete, the efficacy of individual treatments and late pulmonary outcomes could not be determined.

## 1 Introduction

Exogenous lipoid pneumonia (ELP) is a rare pulmonary disorder caused by the inhalation or aspiration of vegetable, animal, or mineral oils, resulting in acute or chronic inflammation of the pulmonary interstitium and parenchyma (1, 2). Its manifestations, including cough, tachypnea, fever, and dyspnea, are nonspecific. CT findings such as consolidation, ground-glass opacities, nodules, and a crazy-paving pattern may overlap with infectious pneumonia and other diffuse lung diseases, making delayed diagnosis common (1). Here, we report a former preterm infant with ELP caused by repeated paraffin oil aspiration and complicated by concomitant *Klebsiella aerogenes* lower respiratory tract infection. This case highlights the importance of repeated targeted history-taking, integrated diagnostic reasoning, and cautious interpretation of treatment response.

**Table 1:** Timeline of the clinical course.

Date	Key clinical findings	Assessment and management
Sep 20–24, 2024	Hospitalized for adhesive intestinal obstruction. Repeated oral liquid paraffin administration with choking was disclosed later.	Conservative management of intestinal obstruction.
Sep 24, 2024	Admitted with cough, fever, and diarrhea; WBC 18.11 × 10⁹/L, CRP 65.54 mg/L, PCT 0.28 ng/mL; bilateral pulmonary opacities; throat-swab tNGS detected K. aerogenes.	Bronchopneumonia and norovirus enteritis initially considered; oxygen, empirical antimicrobials, nebulization, and supportive care.
Sep 28, 2024	Respiratory deterioration with tachypnea and retractions; PCT 0.22 ng/mL; CT showed bilateral consolidation, ground-glass opacities, septal thickening, crazy paving, and focal fat attenuation.	Transferred to PICU; NCPAP, linezolid, meropenem, bronchoscopy, and BAL.
Sep 30, 2024	Persistent fever, cough, tachypnea, and radiological progression. Repeated history-taking revealed multiple paraffin administrations with choking. BALF showed vacuolated histiocytes.	ELP first suspected and clinically diagnosed; paraffin exposure discontinued; corticosteroids, postural drainage, respiratory support, targeted antimicrobials, and empirical adjunctive IVIG continued.
Oct 4–20, 2024	Respiratory status stabilized; no cough or tachypnea at discharge.	Respiratory support downgraded; discharged on October 20.
Oct 30, 2024 (day 32)	Clinically stable without respiratory symptoms; CT showed partial resolution.	Outpatient follow-up.
Dec 3, 2024 (day 66) and Feb 20, 2025 (day 145)	Serial CT showed continued absorption and progressive radiological improvement.	Long-term standardized follow-up later became unavailable after family relocation.

## 2 Case presentation

A 4-month-and-10-day-old boy was admitted on September 24 with a 2-day history of cough and fever and a 1-day history of diarrhea. Initially, he developed a paroxysmal cough with audible sputum in the throat and wheezing, without dyspnea or cyanosis. His maximum body temperature was 38.9 °C. One day before admission, he developed yellow watery stools 5–6 times per day. Symptomatic treatment, including anti-infective therapy, nebulization, and antidiarrheal therapy, did not relieve his symptoms.

He was born at 32 ^+^ ^6^ weeks of gestation by cesarean section because of premature rupture of membranes, with a birth weight of 2.05 kg. His recent medical history was notable for hospitalization from September 20 to September 24, 2024, for adhesive intestinal obstruction, which improved after conservative treatment. There was no history of drug or food allergy. Although aspiration-related history was actively investigated at admission, the caregivers initially did not report any relevant oily substance exposure or choking episodes.

On admission, his temperature was 37.8 °C, heart rate was 128 beats/min, respiratory rate was 32 breaths/min, oxygen saturation was 96%, and body weight was 5.2 kg. He was alert and responsive. Lung auscultation revealed coarse breath sounds bilaterally, with a few fine, moist rales and wheezes at the lung bases. The remainder of the physical examination was unremarkable except for the liver being palpable 3 cm below the right costal margin.

Initial laboratory testing showed a white blood cell count of 18.11 × 10^9^/L, hemoglobin of 101 g/L, platelet count of 475 × 10^9^/L, C-reactive protein of 65.54 mg/L, and procalcitonin of 0.28 ng/mL. Stool viral testing was weakly positive for norovirus, whereas stool routine examination was normal. Chest radiography on September 24 showed patchy bilateral pulmonary opacities. Throat-swab targeted next-generation sequencing (tNGS) detected *Klebsiella aerogenes*.

The patient was initially diagnosed with bronchopneumonia and norovirus enteritis and was treated with nasal cannula oxygen, empirical antimicrobial therapy, nebulization, and supportive care. On September 28, his condition deteriorated, with tachypnea, labored breathing, and inspiratory retractions, and he was transferred to the PICU. Repeat testing showed a procalcitonin level of 0.22 ng/mL and a BNP level of 112.70 pg/mL. Chest CT demonstrated bilateral patchy and confluent consolidations with surrounding ground-glass opacities, interlobular septal thickening, and a crazy-paving pattern (Figures 1A–C). A focal area of fat attenuation (−30 to −150 HU) was identified within a right lung consolidation (Figures 2A,C). Because no relevant oily substance exposure history was available at that time, this finding was not initially recognized as suggestive of ELP.

**Figure 1:**
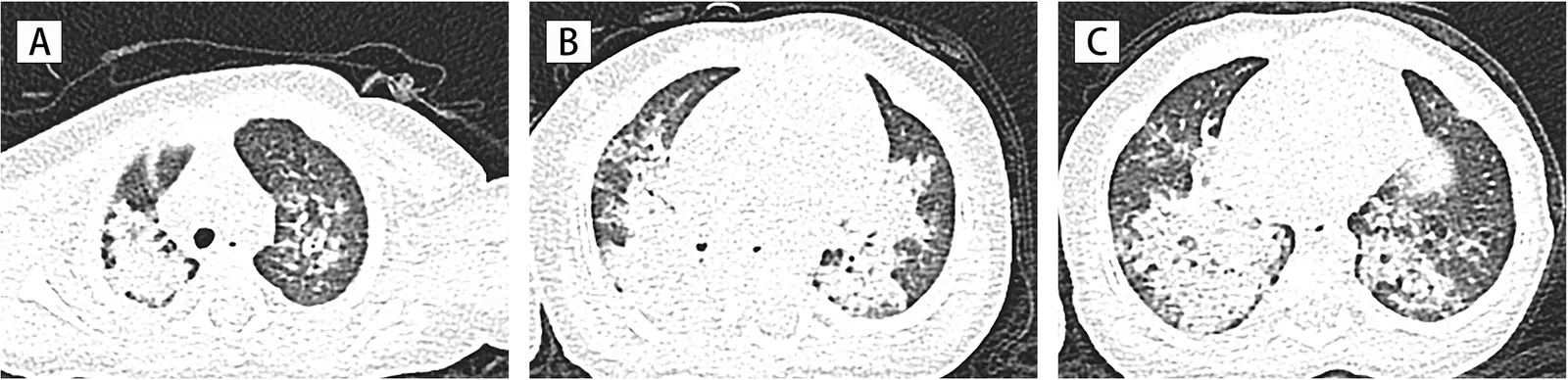
Representative axial lung-window chest CT images obtained at PICU admission. **(A–C)** Bilateral patchy and confluent consolidations with surrounding ground-glass opacities, interlobular septal thickening, and crazy-paving changes are shown at three thoracic levels.

**Figure 2:**
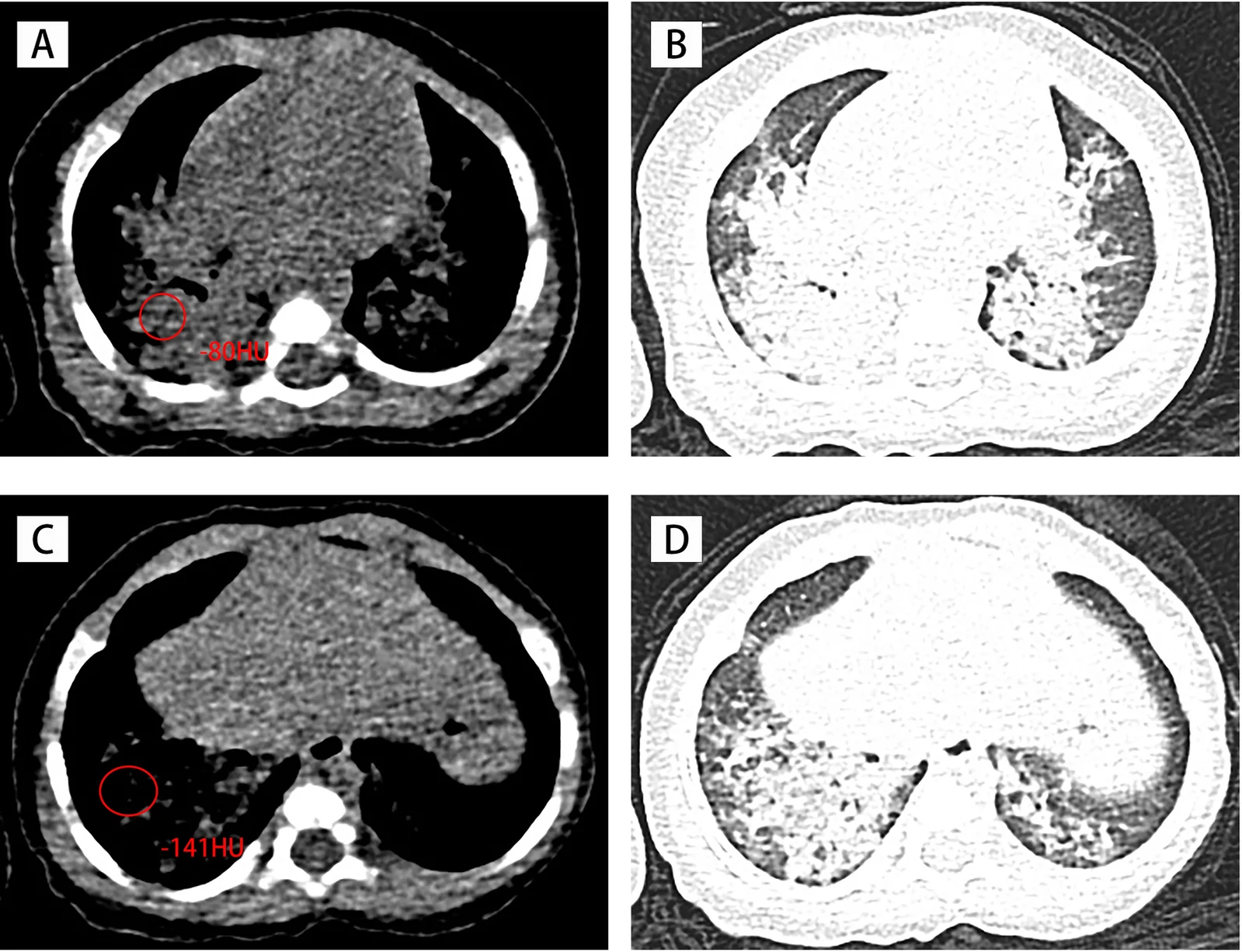
Representative paired chest CT images obtained at PICU admission. **(A,C)** Mediastinal-window images showing focal areas of fat attenuation within the right lung consolidation, with representative ROI attenuation values of −80 HU and −141 HU, respectively. **(B,D)** Corresponding lung-window images at the same anatomical levels, showing bilateral patchy and confluent consolidations with surrounding ground-glass opacities and interlobular septal thickening.

**Figure 3:**
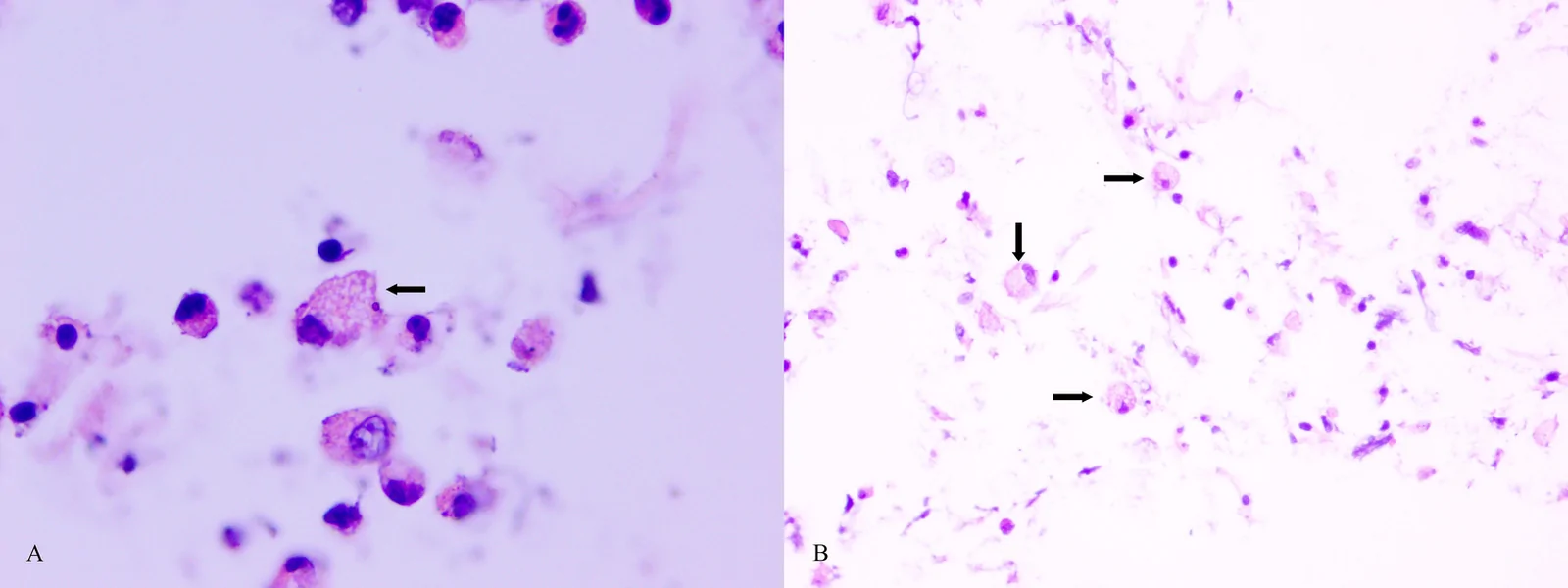
BALF cytology (H&E, ×100). Histiocytes containing clear intracytoplasmic vacuolar structures are indicated by arrows. The morphology was compatible with lipid-containing material but was not diagnostic because lipid-specific staining was unavailable.

Following admission to the PICU, the patient received noninvasive continuous positive airway pressure (NCPAP), linezolid, and meropenem, together with fluid, electrolyte, and acid-base management and bronchoscopy with bronchoalveolar lavage. BALF tNGS also detected *K. aerogenes*, and sputum culture yielded the same organism. Antimicrobial susceptibility testing showed susceptibility to piperacillin-tazobactam, ceftriaxone, cefoperazone-sulbactam, and meropenem, and resistance to cefuroxime, cefuroxime axetil, and trimethoprim-sulfamethoxazole. Meropenem was therefore continued as susceptibility-concordant antimicrobial therapy.

On September 30, persistent fever, cough, tachypnea, and radiological progression prompted repeated targeted history-taking, and ELP was first suspected. The caregivers then disclosed that, during the previous hospitalization for adhesive intestinal obstruction, liquid paraffin had been administered orally multiple times by the caregivers using a spoon while the infant was held upright. The exact dose per administration and total number of administrations could not be reliably reconstructed from the available records and caregiver recall. Brief coughing and choking occurred only during administration; the caregivers regarded these episodes as transient difficulty with oral medication and did not report them to the medical team. Consequently, no targeted aspiration precautions or formal swallowing assessment were initiated at that time. Because the infant was born preterm and had a corrected age of less than 3 months, immature suck-swallow-breathing coordination was considered a potential aspiration risk, although dysphagia was not objectively confirmed.

BALF cytology showed histiocytes containing clear intracytoplasmic vacuolar structures. These findings were compatible with lipid-containing material; however, lipid-specific staining was unavailable, and the cytological findings were interpreted as supportive rather than confirmatory evidence. Based on the repeated paraffin oil exposure with choking, characteristic CT findings, BALF morphology, and clinical course, a clinical diagnosis of ELP complicated by concomitant bacterial lower respiratory tract infection was established.

Treatment was subsequently adjusted to include intravenous methylprednisolone at 2 mg/kg/day, inhaled corticosteroids, NCPAP respiratory support, postural drainage, and continued susceptibility-concordant antimicrobial therapy. Given the severe progressive inflammatory lung injury, concomitant infection, previous prematurity, and very young corrected age, intravenous immunoglobulin (IVIG) was administered empirically at 400 mg/kg/day for 5 consecutive days (total dose, 2 g/kg) as an adjunctive immunomodulatory intervention. During this multimodal treatment, the patient's fever, cough, airway secretions, tachypnea, and respiratory distress gradually improved. By October 4, his vital signs and respiratory status had stabilized, and respiratory support was downgraded to nasal cannula oxygen.

The patient was discharged on October 20, 2024, without cough, tachypnea, or a requirement for respiratory support. At outpatient follow-up on October 30, 2024 (day 32), he remained clinically stable without respiratory symptoms, and chest CT showed partial resolution and decreased density of the multifocal pulmonary opacities. Further CT examinations on December 3, 2024 (day 66), and February 20, 2025 (day 145), demonstrated continued absorption of the pulmonary lesions and progressive radiological improvement (Figure 4).

**Figure 4:**
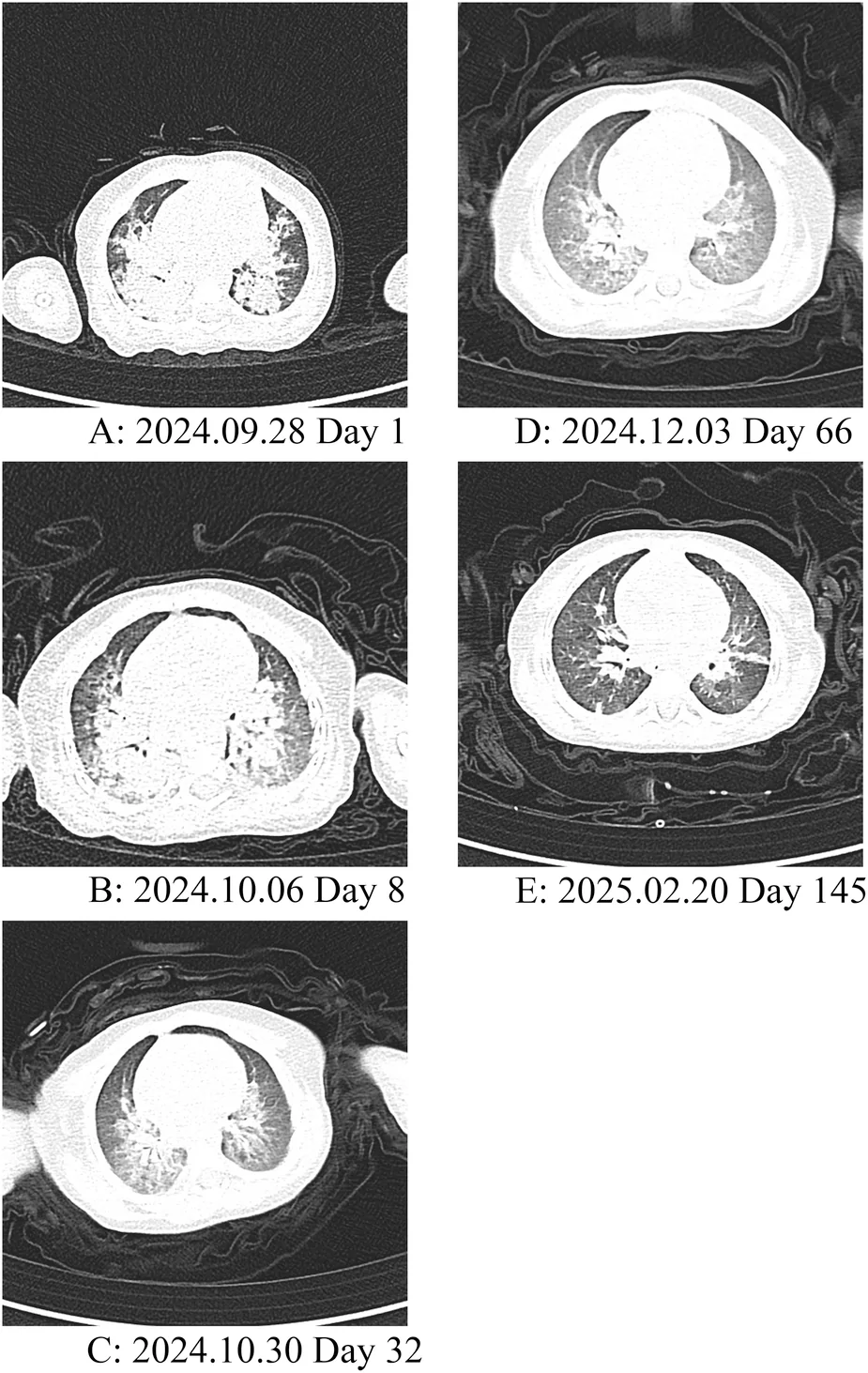
Serial chest CT examinations demonstrating progressive radiological improvement: **(A)** September 28, 2024 (day 1); **(B)** October 6, 2024 (day 8); **(C)** October 30, 2024 (day 32); **(D)** December 3, 2024 (day 66); and **(E)** February 20, 2025 (day 145).

### Guardian perspective

Because the patient was an infant, the perspective was provided by his caregivers. They reported that they initially regarded the brief coughing and choking during paraffin administration as transient difficulty with oral medication and did not recognize these events as possible aspiration; consequently, the events were not reported to the medical team at that time.

## 3 Discussion

Exogenous lipoid pneumonia is a rare noninfectious inflammatory lung disease caused by aspiration or inhalation of exogenous lipids (3, 4). Its presentation is heterogeneous and often overlaps with infectious pneumonia, with cough, sputum production, fever, tachypnea, and dyspnea as common manifestations (1).

The patient's previous prematurity and very young corrected age may have increased the risk of aspiration because of immature swallowing, cough reflexes, and suck-swallow-breathing coordination (5–10). During the previous hospitalization, liquid paraffin was given orally by the caregivers using a spoon while the infant was held upright. Brief choking was not recognized as possible aspiration and was not reported to medical personnel. This case emphasizes that coughing or choking during administration of an oily medication to an infant should prompt immediate discontinuation, assessment of aspiration risk, and notification of medical personnel. Once aspirated, paraffin oil may impair mucociliary clearance and surfactant function and may trigger macrophage-mediated alveolar inflammation and, in persistent cases, fibrosis (11–14).

ELP was first suspected on September 30, 2024, after clinical and radiological progression despite initial antimicrobial therapy. Infectious pneumonia was initially favored because of fever, leukocytosis, elevated C-reactive protein, bilateral pulmonary opacities, and positive respiratory microbiological findings. Although the initial CT showed a focal fat-attenuation area, the absence of a reported oily substance exposure history limited its immediate interpretation. Repeated targeted history-taking subsequently identified paraffin administration with choking. CT manifestations of lipoid pneumonia include consolidation, ground-glass opacities, nodules, interlobular septal thickening, crazy paving, and intralesional fat attenuation. Lesions are often bilateral and multifocal, with middle- and lower-lobe and dependent posterior predominance in aspiration-related cases; peripheral sparing is not a typical or specific finding (1, 15, 16). A solitary fat-attenuation focus is supportive but not independently diagnostic and should be interpreted with the exposure history and other findings.

Several diagnostic limitations should be acknowledged. Lipid-specific staining of BALF, such as Oil Red O or Sudan III staining, was unavailable because of local laboratory limitations. The BALF specimen had undergone routine cytological processing, during which lipid may be dissolved or extracted, and no unfixed residual specimen was available for retrospective staining. Accordingly, the vacuolated histiocytes were considered supportive rather than confirmatory. Lung biopsy was not performed because of its invasiveness and the patient's young age. The diagnosis was therefore clinical, based on the combined exposure history, CT findings, BALF morphology, and clinical course (1, 17–19).

Another important consideration was concomitant bacterial lower respiratory tract infection. *K. aerogenes* was detected by throat-swab tNGS and BALF tNGS and was isolated from sputum culture. The concordant microbiological findings, together with persistent fever, airway secretions, pulmonary moist rales, respiratory deterioration, and bilateral pulmonary opacities, supported clinically significant infection rather than incidental upper-airway colonization. Although procalcitonin levels remained relatively low at 0.28 and 0.22 ng/mL, these values did not exclude bacterial infection in the presence of consistent microbiological and clinical evidence. Prematurity and aspiration-related epithelial injury may also have impaired local pulmonary defense. Clinical and radiological improvement occurred during susceptibility-concordant antimicrobial therapy; however, the relative contributions of bacterial infection and lipid-induced sterile inflammation could not be fully separated (11, 20).

No specific guideline or consensus exists for ELP management, and treatment remains largely empirical (21, 22). The main principles are discontinuation of lipid exposure, respiratory support, airway clearance, and removal of aspirated lipid. Bronchoalveolar lavage may help remove lipid material, lipid-containing macrophages, and inflammatory mediators, whereas whole-lung lavage is technically demanding in infants. Corticosteroids have been used to suppress inflammation, although optimal dose and duration remain uncertain. IVIG is not an established or disease-specific treatment for ELP. In this patient, it was administered empirically at a total dose of 2 g/kg as adjunctive immunomodulatory therapy because of severe progressive inflammatory lung injury, concomitant infection, previous prematurity, and concern regarding relative immune immaturity. Evidence supporting IVIG is limited to observational experience, and its independent contribution cannot be determined because bronchoalveolar lavage, corticosteroids, antimicrobial therapy, respiratory support, postural drainage, and IVIG were administered concurrently (21–23).

Available follow-up through day 145 showed progressive radiological improvement. However, standardized pulmonary function testing, exercise-tolerance assessment, and imaging beyond day 145 were unavailable after the family relocated to another province. Therefore, residual interstitial abnormalities, later fibrosis, or impaired pulmonary function cannot be completely excluded. Other limitations include the absence of lipid-specific staining and histopathological confirmation, the coexistence of infection and sterile inflammatory injury, and the inability to attribute recovery to any single intervention.

In summary, ELP should be considered in infants with treatment-refractory pneumonia, particularly when CT demonstrates intralesional fat attenuation. Detailed and repeated exposure history-taking is essential. In this case, discontinuation of paraffin exposure and multimodal management were associated with clinical and radiological improvement; however, the efficacy of individual interventions, particularly IVIG, cannot be inferred from a single case, and continued long-term follow-up remains important.

## Data Availability

The datasets presented in this article are not readily available because of ethical and privacy restrictions. Requests to access the datasets should be directed to the corresponding author.
